# Nourishment level affects caste-related gene expression in *Polistes* wasps

**DOI:** 10.1186/s12864-015-1410-y

**Published:** 2015-03-25

**Authors:** Ali J Berens, James H Hunt, Amy L Toth

**Affiliations:** Program in Bioinformatics and Computational Biology, Iowa State University, Ames, IA 50011 USA; Department of Ecology, Evolution, and Organismal Biology, Iowa State University, Ames, IA 50011 USA; Department of Biological Sciences, North Carolina State University, Raleigh, NC 27695 USA; Department of Entomology, North Carolina State University, Raleigh, NC 27695 USA; W. M. Keck Center for Behavioral Biology, North Carolina State University, Raleigh, NC 27695 USA; Department of Entomology, Iowa State University, Ames, IA 50011 USA

**Keywords:** Phenotypic plasticity, Nourishment, Social castes, Transcriptomics, *Polistes*

## Abstract

**Background:**

Social insects exhibit striking phenotypic plasticity in the form of distinct reproductive (queen) and non-reproductive (worker) castes, which are typically driven by differences in the environment during early development. Nutritional environment and nourishment during development has been shown to be broadly associated with caste determination across social insect taxa such as bees, wasps, and termites. In primitively social insects such as *Polistes* paper wasps, caste remains flexible throughout adulthood, but there is evidence that nourishment inequalities can bias caste development with workers receiving limited nourishment compared to queens. Dominance and vibrational signaling are behaviors that have also been linked to caste differences in paper wasps, suggesting that a combination of nourishment and social factors may drive caste determination. To better understand the molecular basis of nutritional effects on caste determination, we used RNA-sequencing to investigate the gene expression changes in response to proteinaceous nourishment deprivation in *Polistes metricus* larvae.

**Results:**

We identified 285 nourishment-responsive transcripts, many of which are related to lipid metabolism and oxidation-reduction activity. Via comparisons to previously identified caste-related genes, we found that nourishment restriction only partially biased wasp gene expression patterns toward worker caste-like traits, which supports the notion that nourishment, in conjunction with social environment, is a determinant of developmental caste bias. In addition, we conducted cross-species comparisons of nourishment-responsive genes, and uncovered largely lineage-specific gene expression changes, suggesting few shared nourishment-responsive genes across taxa.

**Conclusion:**

Overall, the results from this study highlight the complex and multifactorial nature of environmental effects on the gene expression patterns underlying plastic phenotypes.

**Electronic supplementary material:**

The online version of this article (doi:10.1186/s12864-015-1410-y) contains supplementary material, which is available to authorized users.

## Background

Phenotypic plasticity provides an important adaptive mechanism by which morphology, physiology, and/or behavior can be adjusted to biotic and abiotic environmental factors including temperature, nutrition, population density, and predator presence [1]. There are a number of striking examples of phenotypic plasticity in insects: dimorphic horn development in dung beetles [2], seasonal color polyphenism in butterflies [3], and wing polyphenism in aphids (reviewed in [4]). One of the best-studied models of insect phenotypic plasticity is reproductive castes in social insects, especially social Hymenoptera (bees, ants, and wasps [5]). In most social insects, genotypic differences do not account for differences between reproductive (queen) and non-reproductive (worker) castes (reviewed in [6]). Instead, environmental factors induce differences in hormone titers and gene expression (reviewed in [6]), leading to the development of queens or workers, which vary in physiology and behavior and for advanced social species, in morphology [5].

One particular environmental factor, food availability, is especially important for caste polyphenism in social insects. Differential nourishment [7-10] and nutrition-related genes and pathways (e.g. storage proteins such as vitellogenin and hexamerin, insulin/insulin-like signaling (IIS) pathways) have been linked to caste differences in honey bees [11,12], paper wasps [13,14], and termites [15,16]. This suggests that the influence of nutrition on caste formation may be broadly shared across diverse taxa [17]. There have been numerous studies investigating the molecular mechanisms underlying queen-worker caste determination in advanced eusocial species, especially honey bees [18-25]. In honey bees, nutritional differences for larvae fed either royal jelly or worker jelly precede a developmental switch resulting in alternative caste phenotypes [26]. Nutrition is also important for caste differences in primitively eusocial species, which lack morphological castes, but we know much less about the molecular mechanisms that underlie the formation of their more subtle behavioral and physiological castes. In primitively eusocial species such as paper wasps, differential nourishment does not strictly determine caste but can lead to a caste bias, whereby female larvae that are fed larger quantities of food are more likely to be future reproductive queens (called “gynes”) as adults [27]. However, the ultimate caste fate of a female is decided during adulthood. First-brood offspring of an established nest are capable of independent reproduction, but instead they perform allomaternal care (worker behavior) as a response to cues emitted by larvae in the nest [28]. Subsequent social reinforcement of worker behavior often occurs via dominance behaviors by the queen or other workers [29,30].

Primitively eusocial taxa such as *Polistes* are an informative group for understanding the evolution of eusociality and the origins of castes [31,32]. Across the annual colony cycle of primitively social wasps in the genus *Polistes*¸ the quantity of larval nourishment changes according to seasonal changes in the adult-to-larva ratio [10,27]. First-brood offspring produced early in the colony cycle have been reared by a single nest-founding queen or few queens, which also perform all colony tasks such as nest building, foraging, egg laying, and brood rearing [30]. At this early time in the colony cycle the adult-to-larva ratio is low [30,33], which leads to low feeding rates and more limited larval nourishment compared to offspring reared by workers and produced later in the colony cycle (future queens or “gynes”). Physiological evidence of nourishment-related differences between workers and gynes collected from naturally-founded colonies in the field include greater fat body stores in gynes [34,35], greater quantities of the storage protein *hexamerin 1* in gynes [36,37], and greater quantities of four additional proteins in gynes [14]. Experimental studies show that nourishment inequalities that correspond to early-season and late-season larval development contexts are associated with development of offspring having characteristics of worker and gyne phenotypes, respectively [38,39].

In addition to the established role of nourishment in caste differences in *Polistes,* social factors, such as dominance behavior [29,31] and maternal influences [40-42] also play a role. Jeanne and Suryanarayanan [40] propose a hypothesis for caste determination in primitively social wasps that incorporates not only nourishment variability, but also social environmental inputs from maternal care, specifically vibrational signals called antennal drumming. Antennal drumming may be an example of a maternal manipulation [43] that directs larvae toward a worker developmental trajectory, and this effect may interact with nourishment induced changes in caste phenotype. Thus, nourishment is likely to act in concert with social environmental factors in determining differences in gyne and worker caste development in *Polistes.*

In this study, we investigated the effect of experimental proteinaceous nourishment deprivation during larval development on caste-related gene expression in a primitively eusocial species, the paper wasp *Polistes metricus*. We had three main goals. First, we explored the transcriptional responses of wasp larvae to high and low nourishment levels during laboratory rearing using RNA-sequencing. Second, we tested the hypothesis that nourishment level relates to caste-related gene expression; specifically that low nourishment is associated with more worker-like gene expression patterns, and high nourishment is associated with more gyne-like expression patterns. To do this, we compared nourishment differential expression to a set of nearly 800 previously identified genes associated with caste development in field-reared *P. metricus* [13], and we did so on multiple levels: individual transcripts, pathways and biological functions. Third, we tested whether the molecular mechanisms underlying the response to nourishment are conserved across taxa by comparing our results to two other nourishment deprivation studies in fruit flies [44] and dung beetles [45]. Our overall goal was to better understand the extent and nature of the role of nourishment and nourishment-related genes in caste development in a primitively social wasp species.

## Results

### Differential expression analysis

**Figure 1:**
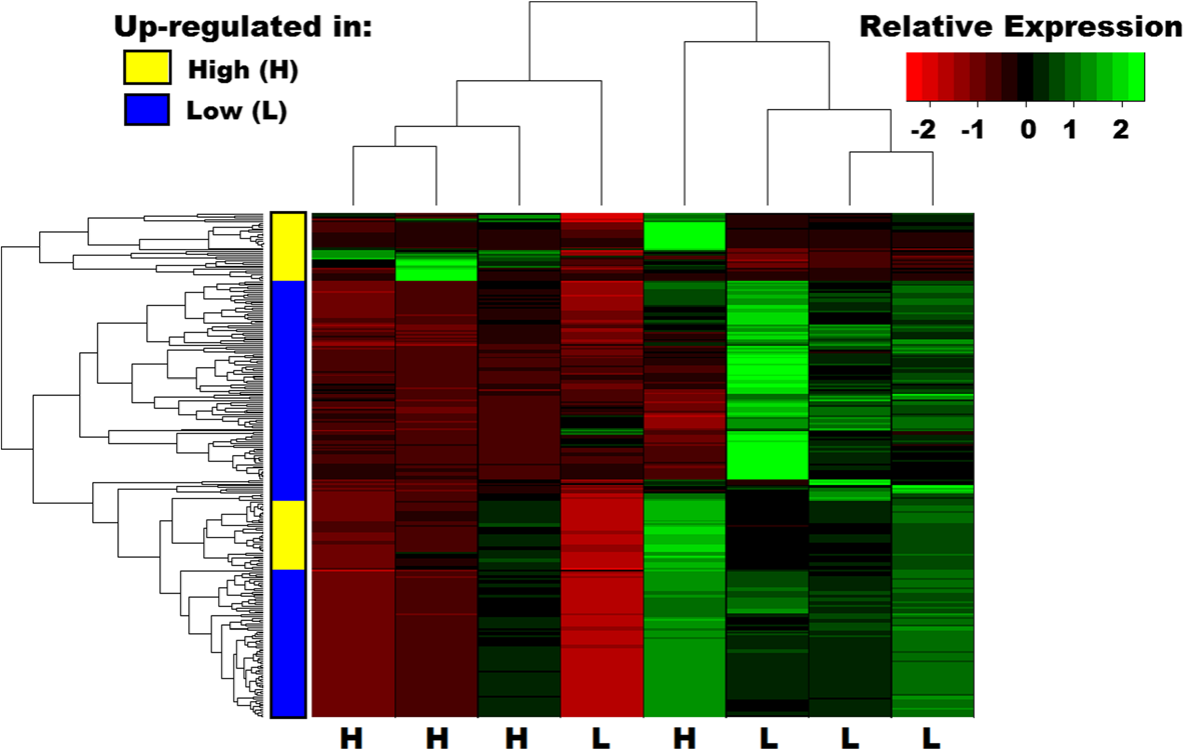
**Heat map of the relative expression (sample read counts scaled by library size then across each gene) for the 284 differentially expressed transcripts between nourishment manipulation levels by sample.** Samples are clustered based on relative expression across all differentially expressed transcripts (top), and transcripts are clustered based on relative expression across all samples (left). Transcripts that are upregulated in high nourishment (H) are highlighted in yellow, and low nourishment (L) upregulated transcripts are highlighted in blue.

To assess differential gene expression, we mapped reads to a previously assembled de novo transcriptome for *P. metricus*; the transcriptome was based on both the sequence data described here in conjunction with additional samples described in a previous study [13]. By comparing expression patterns from head samples from 8 individual wasp larvae under high and low nourishment, we identified 284 *P. metricus* differentially expressed transcripts (DETs) that differed between low and high nourishment treatments, using DESeq (FDR ≤ 0.05, [46]), heretofore referred to as “*P. metricus* nourishment-responsive DETs”. Of these DETs, 207 (72.9%) were upregulated in low compared to high nourishment larvae (Figure 1 is a heat map of the scaled DET read counts across *P. metricus* nourishment samples. Additional file 1 includes the list of *P. metricus* nourishment-responsive DETs). Thus, despite the fact that they had less food available, low nourishment led to a majority of genes having higher gene expression, and thus did not simply cause a general shutdown in transcription.

One important feature of these results is the presence of outlier individuals, i.e. one individual low nourishment larva clustered with the high nourishment samples and a high nourishment larva clustered with the low nourishment samples (Figure 1). The variability of gene expression amongst biological samples is not completely surprising; our previous work has found high inter-individual variation in gene expression [13], effects of lab rearing (JM Jandt, JL Thomson, AC Geffre, AL Toth: Rearing environment may bias social traits: A case study with *Polistes* wasps, submitted), and variable effects of nourishment level on physiology [38].

### Validation of select expression patterns via comparison to qRT-PCR data

It is important to validate RNA-Seq data using another method such as quantitative reverse transcription polymerase chain reaction (qRT-PCR). However, for this study, we had limited samples and quantities of RNA [38], so we could not perform qRT-PCR validation on actual samples from this experiment. In lieu of the sample limitations, we instead made a comparison to pre-existing data on nourishment-responsive expression patterns from adult brains of *P. metricus* under starved vs. ad lib food conditions [47]. Although not ideal because of differences in tissue type and life stage, this comparison still provides a useful point of comparison to validate whether the expression patterns uncovered in this RNA-Seq study are robust.

Of 24 candidate genes, Daugherty et al. [47] identified 10 genes with differential expression in adult *P. metricus* brains reared under low and high nourishment conditions using qRT-PCR. We tested for a correlation (Spearman) in the log_2_ fold changes between the 24 candidate genes from Daugherty et al. [47] and the orthologous transcripts in this study, i.e. best BLAST hits between the primer sequences from Daugherty et al. [47] and the *P. metricus* transcriptome (Additional file 2: Figure S3 is a visual representation of the log_2_ fold changes for both studies; Additional file 3 lists the log_2_ fold change for each gene/transcript and the directionality for differentially expressed genes). For some genes, there is more than one best BLAST hit to the *P. metricus* transcriptome, so all transcripts were used for the correlation analysis. The gene *PmTOR* is absent from the *P. metricus* transcriptome, so this gene was removed from the analysis. Although none of the orthologous transcripts are significantly differentially expressed in the RNA-seq study, we nonetheless identified a significant positive correlation in log_2_ fold changes between these two studies (Spearman ρ: 0.54, p-value = 0.001), which provides support for our observed RNA-Seq results.

One reason we may not have observed statistically significant differential expression of these candidate genes in the RNA-Seq study, despite the strong correlation with the qRT-PCR data, is because of the limited statistical power. Differential expression calls are more stringent in this RNA-Seq study compared to the qRT-PCR analysis because familywise error correction is more severe due to the larger number of genes (over 75,000 compared to 24 genes). Furthermore, the qRT-PCR study has a larger sample size (n = 47) compared to the RNA-Seq study (n = 8), which provides greater statistical power for detecting differential expression.

### Comparison to caste-related gene expression

Next, we investigated how *P. metricus* nourishment-responsive DETs compared to caste-related gene expression. A previous study [13] compared gene expression in field-collected, early season (worker-destined) larvae to late season (gyne-destined) larvae, and identified 736 caste-related DETs. Both low nourishment (described above) and worker-destined larvae [13] show a pronounced bias towards upregulated gene expression (72.9% upregulated in low nourishment and 91.7% upregulated in worker-destined larvae). This pattern generally agrees with the prediction that worker-destined and nourishment-deprived larvae have similar transcriptional states.

**Figure 2:**
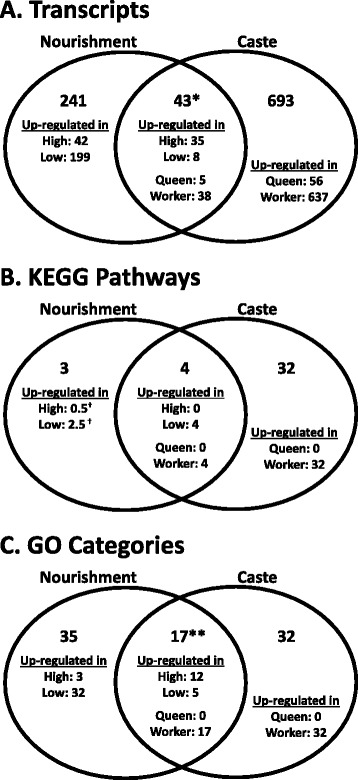
**Number of A) differentially expressed transcripts (DETs), B) KEGG pathways with at least one DET, and C) enriched GO term unique to or shared between nourishment and caste datasets.** Directionality, i.e. upregulated treatment group, is indicated for each dataset, where, for KEGG pathways and GO, terms, directionality is defined as the treatment group with the greater number of upregulated DETs per pathway or category, respectively. * indicates statistically significant overlap between nourishment and caste DETs (Chi-square test with Yates’ correction, p-value < 0.0001). ** indicates statistically significant overlap between nourishment and caste enriched GO terms (Fisher’s exact test, p-value = 4.4e-6). The fatty acid biosynthesis pathway had an equal number of DETs upregulated in the high and low nourishment groups (one per each group), so the directionality of this pathway is counted as one half for each group (indicated by †).

There was a statistically significant but relatively small overlap (43 transcripts) between the nourishment-responsive and caste-related DETs (Chi-square with Yates’ correction: p < 0.0001). Of these shared DETs, the directionality of gene expression change was unexpected: eight (18.6%) were upregulated in low nourishment larvae compared to high nourishment larvae, whereas 38 (88.4%) were upregulated in worker-destined larvae relative to queen-destined larvae (see Figure 2a for a Venn diagram of the number of unique and overlapping caste and nourishment-responsive DETs, Additional file 2: Figure S1 for a heatmap of the overlapping caste and nourishment-responsive DETs, and Additional file 4 for the list of overlapping DETs). To further explore these data beyond examining an overlap of gene lists, we performed a combined statistical analysis of data from both the nourishment level and caste contrasts, and the results also indicate some overlap in gene expression patterns across the two studies (see Methods, with complete description of the approach and results in Additional file 2 and Additional file 5). Taken together, these results partially support our prediction of gene expression similarity between nourishment-responsive and caste-related gene expression but suggest some unanticipated dissimilarities between them.

### Pathway level analysis

Our previous study suggested gene expression similarity across studies is more pronounced on the level of pathways and gene functional categories, rather than specific genes or transcripts [13]. Therefore, we also examined our data at the level of pathways using the Kyoto Encyclopedia of Genes and Genomes (KEGG) [48,49]. Using best BLAST hits to *D. melanogaster*, very few transcripts (799 = 1.0% of the transcriptome) were annotated with enzyme codes for the KEGG analysis with Blast2GO [50] (see Additional file 1 for a list of transcripts annotated with enzyme codes by KEGG pathway). The presence of many transcripts without homology is a shortcoming of the dataset, but is expected and standard for Illumina-based data from non-model species [51]. Only 7% (20) of the nourishment DETs were known members of KEGG pathways, therefore we identified very few (7 of 118) KEGG pathways with at least one DET. We then looked to see if these seven pathways were also related to caste differences [13]. Four of the seven pathways also had caste-related DETs (including glycerolipid metabolism, nitrogen metabolism, and purine metabolism; listed in Additional file 4), but this amount of overlap was not statistically significant (Fisher’s exact test, p-value = 0.12). The majority of the DETs are upregulated in both low nourishment and worker-destined larvae for all four of these shared KEGG pathways (Figure 2b is a Venn diagram of the number of KEGG pathways with nourishment and/or caste DETs), which is in agreement with the prediction that nourishment-responsive biochemical pathways are regulated in a similar direction to what is found between castes in paper wasps.

### Gene ontology (GO) enrichment analysis

**Figure 3:**
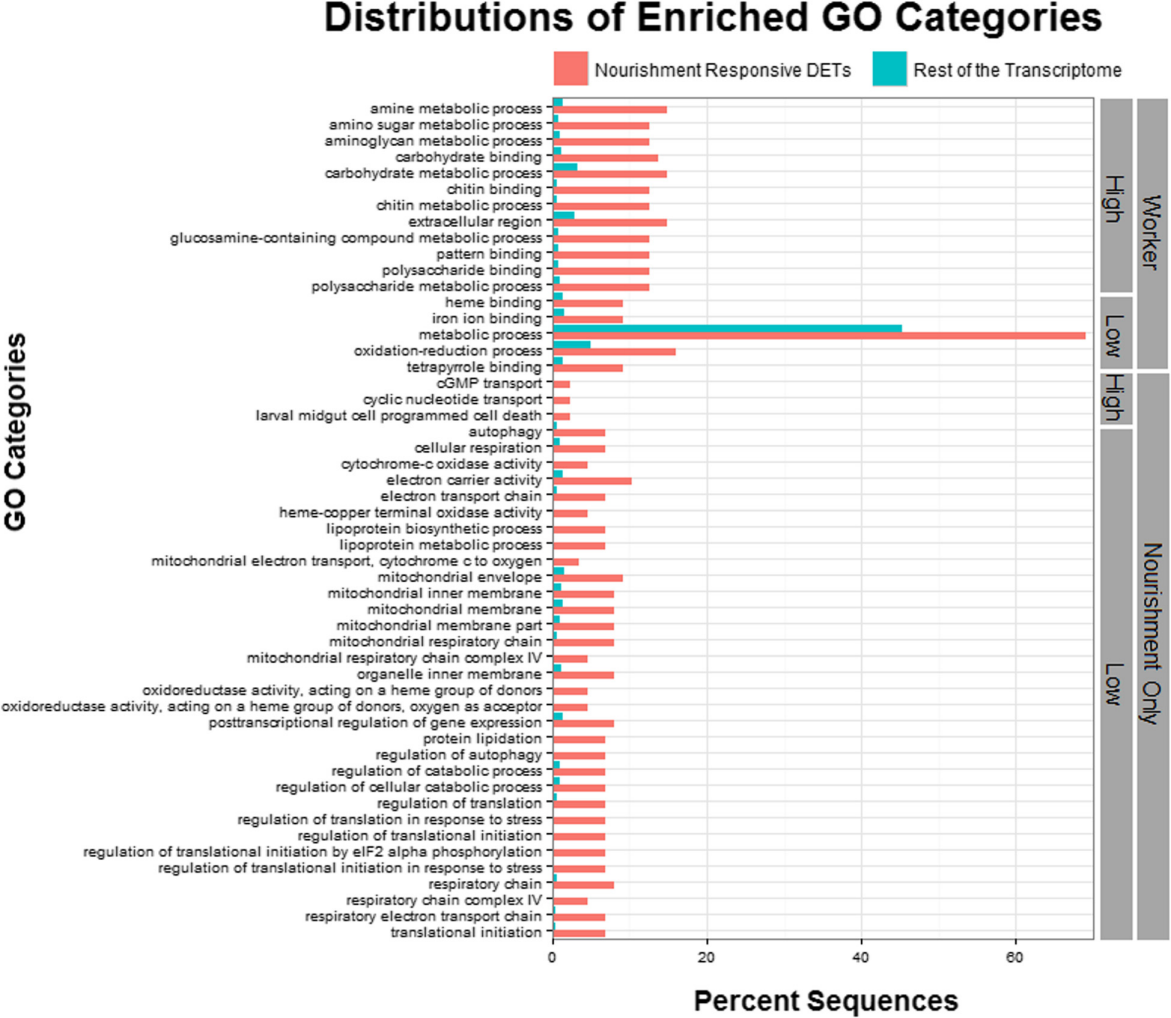
**Bar chart of GO categories significantly enriched (FDR < 0.05; one-tail) between nourishment differentially expressed transcripts (DETs) and remaining transcriptome.** All significantly enriched GO categories were over represented in the nourishment-responsive DETs compared to the rest of the transcriptome. 17 significantly enriched GO categories were shared in common for both caste and nourishment. Directionality is indicated for the enriched GO categories and defined as the treatment group with the greater number of upregulated DETs per category.

On the level of gene functional categories, fifty-two GO terms were significantly enriched within the *P. metricus* nourishment-responsive DETs compared to the remainder of transcriptome (Figure 3; Additional file 1 includes the list of enriched GO terms). These included functions related to lipoprotein metabolism, oxidation reduction activity, and polysaccharide metabolism. More than 30% of GO terms (including terms related to oxidoreductase activity and carbohydrate metabolism) were common to both caste- and nourishment manipulation-related DETs, representing a significant overlap (Chi-square test with Yates’ correction, p < 0.0001; Figure 2b; Additional file 4 lists the shared caste and nourishment enriched GO terms). However, if we examine the direction of differential expression of DETs associated with these GO terms, we do not consistently see the predicted pattern of the same directional bias to both low nourishment larvae and worker-destined larvae (Figure 2b; Additional file 4 lists the direction).

### Cross-species comparisons

Because nourishment is an important driver of phenotypic plasticity in many species, we were interested in determining whether the molecular mechanisms underlying the response to nourishment identified in paper wasps are conserved in other taxa. After an extensive literature search, we identified two studies from other insects that also examined transcriptional responses to nutritional stress: 1) a microarray study of tissue-specific (fat body and muscle) gene expression derived from fruit fly larvae [44] and 2) a microarray study of thoracic horn development in female dung beetle pupae [45]. Although not ideal comparisons to our paper wasp dataset because of differences in life stages and sampled tissues, these studies still provide useful preliminary comparisons to begin addressing whether there are any conserved of nourishment-responsive transcripts.

**Figure 4:**
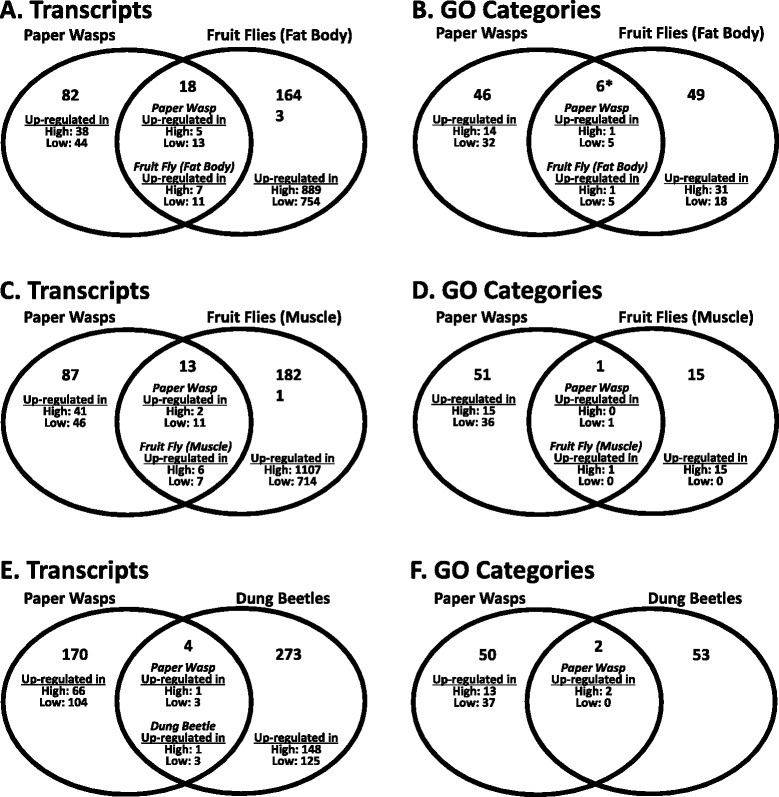
**Number and overlap of nourishment-responsive differentially expressed transcripts (DETs) between** ***Polistes metricus*** **(paper wasp) and A)** ***Drosophila melanogaster*** **(fruit fly) fat body, C) fruit fly muscle, or E)** ***Onthophagus taurus*** **(dung beetle) female thoracic horn.** Number and overlap of of enriched GO terms between paper wasps and B) fruit fly fat body, D) fruit fly muscle, or F) dung beetle thoracic horn. Directionality, i.e. which nourishment treatment group showed upregulation, is indicated for each dataset. There is only a statistically significant overlap between paper wasp and fruit fly wild-type fat body enriched GO terms (indicated by *, Chi-squared test with Yates’ correction, p-value = 9.557e-11). All other comparisons were not significant (Chi-squared tests with Yates’ corrections, p-values > 0.05).

When comparing *P. metricus* nourishment-responsive transcripts with nourishment-responsive transcripts in *D. melanogaster* fat body and muscle tissues [44], we identified small, non-significant overlaps: only 18 and 13 common DETs, respectively (Figure 4a and c; Chi-square tests with Yates’ correction; fat body: p-value = 0.84; muscle: p-value = 0.19). For both tissue types in fruit flies, most transcripts are down-regulated with low nourishment (Additional file 6 lists the common DETs and directionality for each species). Of these shared DETs, the majority are expressed in the same direction in both species: 56% (10 DETs for the fat body dataset; Fisher’s Exact Test, p-value = 0.676) and 54% (7 DETs for the muscle; Fisher’s Exact Test, p-value = 0.730).

At the level of GO categories, there is a statistically significant overlap (six GO terms including carbohydrate metabolic processes [GO:0005975] and oxidation-reduction process [GO:0055114]; see Additional file 6 for the complete list of overlapping GO categories) in the GO terms associated with nourishment-responsive transcripts for *P. metricus* and *D. melanogaster* fat bodies (Figure 4b; chi-squared test with Yates’ correction, p-value = 9.557e-11). The consistency in directionality between shared *P. metricus* and *D. melanogaster* fat body GO categories (i.e. up-regulation in low nourishment samples for all GO categories, except carbohydrate metabolism with up-regulation in high nourishment samples) further supports common functional changes related to nourishment manipulation in both species. However, this signal is not observed in the comparison between *P. metricus* nourishment-enriched GO terms and *D. melanogaster* nourishment-enriched GO terms in the muscle tissue. There is only one common nourishment-enriched GO term (oxidation-reduction process [GO:0055114]; Chi-square test with Yates’ correction, p-value = 0.4235), which is upregulated in the *P. metricus* low nourishment larvae but upregulated in the high nourishment samples for the *D. melanogaster* muscle tissue (Figure 4d).

Out of 18,061 homologous transcripts between *P. metricus* and a dung beetle *Onthophagus taurus* [45], we again identified a very small overlap (4 transcripts; not significant; Chi-square test, p-value = 0.57) in the nourishment-responsive transcripts for *P. metricus* female larvae and nourishment-responsive transcripts in *O. taurus* female pupal thoracic horns. However, this overlap between paper wasps and dung beetles is again consistent in the directionality of nourishment-responsiveness with shared DETs being upregulated in low nourishment individuals (Figure 4e; Additional file 6). Comparing enriched GO terms between paper wasps and dung beetles, we found only two shared GO categories: aminoglycan metabolic process (GO:0006022) and chitin metabolic process (GO:0006030) (Chi-square test with Yates’ correction, p-value = 0.08; Figure 4f), which suggests few common functional changes related to nourishment in samples from these two species.

## Discussion

In this study, we provide the first genome-wide transcriptional profiling of nourishment response during development in the genus *Polistes*, a model for understanding the evolution of social castes. We identified 285 nourishment differential expression transcripts (DETs) between larvae raised experimentally on low vs. high nourishment, many of which are associated with lipid metabolism and oxidation-reduction activity. Most (73%) of the *Polistes* nourishment-responsive transcripts are upregulated in larvae with low nourishment, which is opposite to the pattern observed in other insects including fruit flies [44] and dung beetles [45]. There were few conserved nourishment-responsive transcripts across species, and these were related to aminoglycan metabolism, carbohydrate metabolism, and oxidation-reduction activity. Among transcripts corresponding to those functions, our data show some cross-species consistency in the direction of expression in response to nourishment deprivation. The overall picture from our preliminary cross-species comparisons is that it is largely different genes that show transcriptional responses to nourishment stress across insect orders. However, it is important to note that transcriptional similarities across these three systems may be underestimated from our analysis because of inconsistencies in the datasets such as analyzing different life stages and tissue types. Further work with directly comparable datasets is needed in order to better understand the extent of conservation of nourishment-responsive gene expression across taxa.

Nourishment inequalities have long been considered the most important environmental determinant of castes in *Polistes* [8,14,27,39]. If nourishment plays a major role in caste bias in *P. metricus*, we predicted that worker-destined larvae from natural nests in the field would have similar transcript expression patterns as experimental low nourishment larvae. With respect to directionality of overall transcript expression, this was the case — most DETs were upregulated in worker-destined larvae (92.1%) and in low nourishment larvae (72.9%). This work agrees with previous work in honey bees that also showed more genes with worker-biased expression in larvae, but more genes with queen-biased expression later in development (pupal stage) [18,21]. We also found a small but statistically significant overlap (43 common transcripts) between nourishment-responsive and caste-related DETs in *Polistes* (Chi-square with Yates’ correction: p < 0.0001; Figure 2; Additional file 2: Figure S1). Focusing on the 43 common DETs, however, shows that the majority of these were upregulated in worker-destined larvae but down-regulated in low nourishment larvae, which is opposite to our prediction and to the pattern of all DETs. This suggests that while nourishment restriction in the laboratory may lead to upregulation of transcript expression in some of the same pathways that are related to worker development, this manipulation did not succeed in causing a full shift to worker-like transcript expression patterns.

There was also a significant overlap in nourishment-responsive and caste-related expression at the GO functional level, with terms related to metabolism, protein binding, and oxidation reduction activity (Figures 2 and 3; Additional file 2: Figure S2; Additional file 4). On the level of pathways, we identified a few shared KEGG pathways between nourishment and caste datasets (Figure 2; Additional file 4), which may be due to the low annotation rate of nourishment-responsive DETs. Although there is limited overlap at the level of KEGG pathways, we did observe the expected pattern of directionality in gene expression, with the majority of DETs upregulated in low nourishment and worker-destined larvae for all pathways.

Overall, our data suggest that nourishment level caused a partial shift in gene expression, with low nourishment individuals being more worker-like and high nourishment individuals being more gyne-like. However, it is pertinent to note that the nourishment manipulation may not have resulted in a strong nutritional stress; i.e., wasps may have compensated for low proteinaceous food availability by consuming more sugar. In a recent study examining adult wasps reared from the same nests as the larvae analyzed here, the nourishment manipulation caused only a partial shift in caste-related physiology [38]. In that study, total lipid and protein hemolymph levels in adults were not affected by low nourishment, whereas adults that had been reared with low nourishment showed greater ovary development after two weeks of being fed while in isolation. This counterintuitive observation corresponds to previous studies showing that adult workers collected in the field have greater ovary development than better-nourished gynes [52] and substantial evidence that workers are in a physiological state ready to reproduce upon emergence [38], whereas gynes are in reproductive diapause until after the overwintering period [10]. Taken together, the physiological data [38] and transcriptomic data (this study) indicate that nourishment alone did not completely shift the developmental trajectory of larvae towards one caste or another.

One important caveat of our study is that the effects of the nourishment treatment on gene expression and physiology may be influenced by laboratory rearing, which may produce conflicting results compared to a natural field setting (JM Jandt, JL Thomson, AC Geffre, AL Toth: Rearing environment may bias social traits: A case study with *Polistes* wasps, submitted). Lab-reared wasps typically have higher lipid stores perhaps due to both overfeeding and inactivity [47], and lab-rearing can perturb caste-related gene expression (JM Jandt, JL Thomson, AC Geffre, AL Toth: Rearing environment may bias social traits: A case study with *Polistes* wasps, submitted). Two genes (*inositol oxygenase* and *Hsp90alpha*) known to exhibit caste-specific gene expression are perturbed due to lab rearing, but neither gene was differentially expressed in the current study. In addition, the low and high nourishment samples in our study were reared by single foundresses with unrestricted access to sucrose as opposed to the two feeding levels for caterpillars that they fed to larvae. Diets with a high carbohydrate to protein ratio can cause an increase in lipid levels in the insect fat body [53-55]. The effects of lab-rearing could thus be a factor in some of the non-overlap we observed between nourishment-responsive and caste-related DETs in the current study. Nonetheless, our data do suggest that low nourishment can trigger expression of some of the same genes and pathways that are associated with worker caste development in *Polistes metricus.*

Our results provide insight into the role of nourishment in the differential gene expression patterns that lead to different castes in *Polistes*. Our results also support the notion that nourishment inequalities alone cannot explain all caste variability in gene expression. Instead, caste-related gene expression bias is likely to be additionally influenced by social factors such as dominance behavior [29,31], vibrational communication [40-42], and/or epigenetically mediated environmental influences [56]. Further work on gene expression in relation to nourishment, other influences on caste determination in *Polistes,* and interactions among them can further advance our growing understanding of caste determination in primitively social wasps.

## Conclusions

This study provides new data on nourishment-responsive gene expression in the context of caste development for *Polistes metricus*, a model for studying the evolution of social insect castes. We identified suites of nourishment-responsive transcripts in developing *P. metricus* larvae. Interestingly, most transcripts were upregulated when larvae experienced proteinaceous nourishment deprivation; thus, reduced food level did not shut down gene expression but instead resulted in active transcription of many genes including several involved in lipid metabolism, carbohydrate metabolism, and oxidation-reduction processes. By comparing to previously reported caste-related gene expression patterns from the same species, we uncovered some similarity in transcripts, pathways, and gene functions related to both nourishment deprivation and worker caste-biased expression. However, many caste-associated genes were not found to be nourishment-responsive, so there are additional factors (likely including many social environmental factors) that influence caste-related gene expression. These results support the notion that nourishment level during development can somewhat bias development into queen or worker caste as adults, but leave room for other factors, and thus underscore the complex and multifactorial nature of caste development.

## Methods

### Samples

We collected adult female *P. metricus* wasps in early March 2009 near Raleigh, NC, as they were exiting the attic of a house in which they had passed the winter in behavioral quiescence and reproductive diapause. Single wasps were placed in cages 30 cm in length, width, and height constructed of clear plastic with plastic screen on the top and two sides. An opening in the top was covered with a piece of hardboard 10 cm square with a nest from a previous season attached to the underside. Nests were trimmed to seven cells ca. 0.5 cm deep, and meconia (larval feces) of the original nest occupants were removed. Each seven-cell nest therefore served as a “starter” nest on which the newly-captured wasps could initiate construction and then expand as their own. Construction paper was the pulp source for nest construction.

Caged wasps were placed on wire racks in a growth chamber 1.2 m × 2.5 m × 2.1 m in the North Carolina State University Phytotron. The chamber contained incandescent lights on a 16 L/8D cycle; fluorescent lights came on 2 h following and went off 2 h before the incandescent lights. Light intensity was 21 micromoles/sec/m^2^ in the incandescent-only morning and evening periods and 225 micromoles/sec/m^2^ during the both-lights midday. Chamber temperature was 20°C at night and 30°C during full light, with a gradual ramp-up in the morning and ramp-down in the evening. Each day, we repositioned cages in a rotation pattern to distribute any light or temperature variation in the chamber across all colonies over the course of the experiment.

Each cage was provided with ad libitum sucrose (rock candy) and a water source (15 ml tube plugged with cotton). Both high and low nourishment groups had equal access to sucrose, but differed in the amount of proteinaceous food provided. Proteinaceous nourishment was provided in the form of late 3^rd^ or early 4^th^-instar *Manduca sexta* larva ca. 2.0 cm in length. Low nourishment foundresses received one caterpillar every fourth day. After May 22, the feeding schedule for low nourishment foundresses was accelerated to one caterpillar every three days. In the high nourishment treatment, foundresses were provided with caterpillars *ad lib*, adjusted each day to one caterpillar above the number that had been consumed since the previous day’s provisioning. For this experiment, we collected the largest final instar larva in each nest immediately following spinning of a cocoon by the third larva in the nest to do so. Those three larvae were allowed to develop into adults that then were analyzed for developmental and physiological characteristics. These results were published separately [38]. For our study, four larvae per treatment were flash frozen in liquid nitrogen and stored at -80°C prior to RNA extraction.

### RNA-extraction and RNA-sequencing

*Polistes* possess behaviorally distinct, not morphologically distinct castes, thus we were especially interested in developmental expression changes in larval brains. This led us to choose larval heads as the target tissue for our analysis. Whole heads were used rather than whole brains because of concerns about larval brain dissection quality and low RNA yields. We acknowledge that it is likely that the nourishment manipulation caused additional gene expression changes in other tissues we did not sample. However, we did not use whole bodies for two reasons: 1) our primary interest in brains and behavioral castes, and 2) the fact that brain gene expression differences in social insects can be subtle [57], meaning that high levels of expression from the fat body and other tissues could have drowned out expression patterns from the brain.

To preserve RNA during dissection, we removed the head region (indicated by dark coloration) of each individual larva, while kept on dry ice, using a sterilized razor blade. From these individual larval heads, we extracted total RNA using an RNeasy Mini Kit (Quigen), which was quality controlled using spectrophotometry (NanoDrop 2000) and a Bioanalyzer (Agilent). The High-Throughput Sequencing and Genotyping Unit of the W.M. Keck Center (University of Illinois at Urbana-Champaign) prepared 16 mRNA Seq libraries (n = 4 per group, high and low nourishment level) derived from two experimental nourishment levels (high and low; this study) and unmanipulated castes (queen- and worker-destined; [13]) using the “TruSeq RNAseq Sample Prep kit” (Illumina). These prepared libraries were sequenced on a HiSeq 2000 (Illumina) to generate over 1 billion 100 base paired-end reads, which we assembled *de novo* into a transcriptome (deposited at DDBJ/EMBL/GenBank under the accession GBGV00000000; this study used the first version GBGV01000000). Raw sequence data has been deposited to the National Center for Biotechnology Information’s (NCBI) Short Read Archive (BioProject ID: PRJNA242774, [accession numbers: NCBI:SRX511425, NCBI:SRX511426, NCBI:SRX511427, NCBI:SRX511430, NCBI:SRX511432, NCBI:SRX511433, NCBI:SRX511434, and NCBI:SRX511435]). For a more details about the RNA-Sequencing and the transcriptome assembly, see [13].

### Read mapping, abundance estimation, and differential expression analysis

As described in [13], we aligned the raw paired-end reads to the reference transcriptome (GBGV01000000) using Bowtie 2 (Version 2.1.0) [58] with default settings. From these alignments, we quantified transcript abundances for each library using eXpress (Version 1.3.1) [59], which have been depositied in NCBI’s Gene Expression Omnibus [60] and are accessible through GEO Series [NCBI:GSE61960] (http://www.ncbi.nlm.nih.gov/geo/query/acc.cgi?acc=GSE61960). From these raw read counts, we identified differentially expressed transcripts (FDR ≤ 0.05, [46]) between nourishment manipulation levels (high and low) using the R (Version 3.0.1) [61] statistical package DESeq (Version 1.12.0) [62] downloaded from the Bioconductor repository [63]. Raw read counts were normalized by the effective library size, and dispersion factors were determined based on the per-condition method.

### Kyoto encyclopedia of genes and genomes (KEGG) and gene ontology (GO) analyses

From functional annotations based on best BLASTx hit (E-value ≤ 1e-3) to *Drosophila melanogaster* sequences, we used Blast2GO (Version 2.6.5) [50] to assign enzyme codes, metabolic (KEGG) pathways, and GO terms to *P. metricus* transcriptomic sequences (see [13]). Based on these assignments, we identified the KEGG pathways that contain nourishment DETs and assessed enrichment of GO terms (FDR ≤ 0.05; one-tailed, [46]) between the nourishment DETs compared to the background – the complete *P. metricus* transcriptome.

### Caste and nourishment manipulation comparison

To assess the extent that differential nourishment biased gene expression patterns toward a particular caste phenotype in *Polistes* paper wasps, we compared molecular signatures of nourishment manipulation and caste determination [13] at three levels: transcript differential expression, KEGG pathways with DETs, and GO enrichment. At the transcript level, we tested for significance in overlap between nourishment and caste DETs given the number of DETs per each dataset and the total number of *P. metricus* transcripts by using a Chi-square test with Yates’ correction. For both the nourishment and caste datasets, we identified which KEGG pathways had at least one DET, and we then performed a Fisher exact test to determine whether there is a statistically significant number of shared KEGG pathways with DETs given the number of unique pathways with DETs per each dataset and all pathways without DETs. Finally, we assessed significance in the overlap between nourishment and caste GO enriched categories in relation to the total number of annotated, not enriched, GO terms and unique nourishment/caste enriched GO terms using a Fisher exact test.

### Alternative statistical approach to address overlap across nourishment and caste studies

We investigated an alternative approach (beyond comparisons of DET lists) to test for similarity in the transcript expression patterns associated with nourishment restriction and worker-caste development. In some sense, across the two studies we have a replicated experiment with respect to nourishment levels, which utilizes both the lab and field setting. As described in the introduction, worker-destined typically larvae receive more limited nourishment compared to queen-destined larvae [10,27,30,33]. Therefore, we modeled these datasets together while controlling for location (lab or field) to identify nourishment-responsive transcripts. Overall, our results suggest some consistency in the modeling and list comparison approaches. For full details and results using this approach, please see Additional file 2 for Supplemental Methods, Results, Table, and Figures S4-S7 and Additional file 5 for lists of nourishment-responsive transcripts.

### Cross-species comparison

Based on our interest in whether nourishment differences induce similar molecular responses in transcript expression and biological processes, we reviewed the literature and searched data repositories (NCBI Gene Expression Omnibus and EMBL ArrayExpress) to identify the most directly comparable studies to the *P. metricus* nourishment manipulation dataset based on the following criteria: 1) the datasets compared late-stage subadult insects reared on two nourishment levels and 2) the studies used a transcriptome-wide approach, i.e. microarray or RNA-seq. We identified two studies that meet both of these criteria: one in fruit flies (*D. melanogaster*, [44]) and the other in dung beetles (*Onthophagus Taurus*, [45]). For this analysis, we compared lists of putative orthologous nourishment-biased DETs and nourishment enriched GO terms between paper wasps and fruit flies or dung beetles using Chi-square tests with Yates’ correction. We defined putative orthologous sequences between *P. metricus* and either *D. melanogaster* or *O. taurus* as the best BLAST hit (E-value ≤ 1e-3) between pairs of species. For the fruit fly comparison, we only used the wild-type fruit fly data, and we compared the paper wasp dataset to both tissue types in fruit flies (adipose and muscle). The GO terms listed in the publication of Teleman et al. [44] were not consistent with the current Gene Ontology database, so we identified fruit fly nourishment enriched using Blast2Go (FDR ≤ 0.05; one-tailed, [46]). For the dung beetle comparison, we focused our study on a comparison with female thoracic horn dataset, which we felt was the most akin to the paper wasp dataset because samples were derived from tissues in female insects during the pupal stage of development.

### Availability of supporting data

Raw sequence data has been deposited to the National Center for Biotechnology Information’s (NCBI) Short Read Archive (BioProject: PRJNA242774, http://www.ncbi.nlm.nih.gov/bioproject/?term=PRJNA242774). The transcript abundance data is available in the NCBI’s Gene Expression Omnibus repository (Accession number: GSE61960, http://www.ncbi.nlm.nih.gov/geo/query/acc.cgi?acc=GSE61960). All other data sets supporting the results of this article are included within the article and its additional files.

## Additional files

Additional file 1:
***Polistes metricus*** **differentially expressed transcripts, KEGG pathways, and GO categories.** Contains the results of the differential expression analysis on the nourishment data, the list of KEGG pathways with more than one differentially expressed transcript, and the list of nourishment enriched GO categories. Additional file 2:
**Supplemental methods, results, tables, and figures.** Includes supplemental figures for the overlap analysis of nourishment and caste and the results of the validation by comparison to qRT-PCR data. This file also contains the supplemental methods, results, tables, and figures of the additional statistical approach for determining overlap between the nourishment and caste data sets. Additional file 3:
**Validation by comparison to qRT-PCR data.** Contains the log_2_ fold changes for the homologous sequences between the RNA-seq and qRT-PCR datasets. Sequences that are not differentially expressed are listed as NDE; whereas, the directionality is listed for the differentially expressed transcripts. Additional file 4:
**Results of nourishment and caste comparison at levels of transcripts, pathways, and biological functions.** Lists the common differentially expressed transcripts, KEGG pathways, and GO categories between the nourishment and caste data sets. Additional file 5:
**Results of additional statistical approach for determining overlap between nourishment and caste data sets.** Includes the estimates of nourishment main effects and nourishment by location interaction effects for the transcripts with significant effects. Additional file 6:
**Nourishment comparison across species.** Lists the overlapping nourishment differentially expressed transcripts and GO categories for the comparisons between paper wasps and fruit flies (fat body and muscle tissue) or dung beetles.

## Abbreviations

DETs: Differentially expressed transcripts
FDR: False discovery rate
GO: Gene ontology
KEGG: Kyoto encyclopedia of genes and genomes
NCBI: National Center for Biotechnology Information
qRT-PCR: Quantitative reverse transcription polymerase chain reaction
