# Supplementary material for: Nourishment level affects caste-related gene expression in Polistes wasps
Source: BMC Genomics. 2015 Mar 25;16(1):235. doi: 10.1186/s12864-015-1410-y (PMC4381360; doi:10.1186/s12864-015-1410-y)
Supplement: Additional file 2: — Supplemental methods, results, tables, and figures. Includes supplemental figures for the overlap analysis of nourishment and caste and the results of the validation by comparison to qRT-PCR data. This file also contains the supplemental methods, results, tables, and figures of the additional statistical approach for determining overlap between the nourishment and caste data sets. [file 12864_2015_1410_MOESM2_ESM.docx]

**Supplemental Methods**

In addition to comparing lists of differentially expressed transcripts between nourishment treatment (this study) and caste [[1](#_ENREF_1)](Berens et al. 2014) separately as described in the main text, we also modeled the two datasets together. Because worker-destined larvae are known to have lower levels of nourishment than gyne-destined larvae, we actually have the opportunity to model the effects of differential nourishment in both settings by combining the data from both studies. From our previous study, which used samples collected directly from the field, four samples were collected early in the season when the larval nourishment is low due to small adult-to-larvae ratio and four samples collected late in the season when larval nourishment is high because of a high adult-to-larvae ratio [[1](#_ENREF_1)](Berens et al. 2014). Then, we can use the location of the samples (lab or field) as the blocking factor where each block contains eight samples with four assigned to each nourishment level. In the model, we will control for the blocking factor in order to identify transcripts with significant nourishment effects and nourishment by location effects. The model is described below.

Model

Let $Y_{ijkt}$ denote the read counts of block (location) $i=\{field, lab\}$, nourishment level$j=\{low, high\}$, and replicate $k=\{1,2,3,4\}$ for transcript $t=\left\{ 1,2,\ldots,74516 \right\}$. Then the full main effects and interaction model for $Y_{ijkt}$ is:

$$Y_{ijkt}=\mu_{\ldots t}+\beta_{i..t}+\tau_{.j.t}+\left( \beta\tau\right)_{ij.t}+\epsilon_{ijkt}$$

where $\mu_{\ldots t}$ is the mean read count (expression), $\beta_{i..t}$ is the main effect of the $i$th block (location), $\tau_{.j.t}$ is the main effect of the $j$th nourishment level, $\left( \beta\tau\right)_{ij.t}$ is the interaction effect for the combination of location $i$ and nourishment level $j$, and $\epsilon_{ijkt}$ is the random residual effect for the $k$th replicate of the $j$th nourishment level in the $i$th location for transcript $t$. The model is over-parameterized, so we impose the following constraints: $\beta_{i=field..t}=0$, $\tau_{.j=high.t}=0$, $\left( \beta\tau\right)_{i=fieldj.t}=0$ for all $j$, and $\left( \beta\tau\right)_{ij=high.t}=0$ for all $i$.

Test for Nourishment Effect

The nourishment-responsive transcripts are identified as the transcripts where the null hypothesis that expression under low and high nourishment is equivalent:

$$H_{0}:\tau_{.j=low.t}=0$$

is rejected (FDR ≤ 0.05, [[2](#_ENREF_2)]) in favor of the alternative hypothesis that there is differential expression between nourishment levels:

$$H_{0}:\tau_{.j=low.t}\neq0.$$

To do this, we contrast the reduced location only effects model:

$$Y_{ijkt}=\mu_{\ldots t}+\beta_{i..t}+\epsilon_{ijkt}$$

with the reduced location and nourishment effects model:

$$Y_{ijkt}=\mu_{\ldots t}+\beta_{i..t}+\tau_{.j.t}+\epsilon_{ijkt}$$

using the generalized linear model test in DESeq [[3](#_ENREF_3)] with pooled dispersion. From this test, we have a list of nourishment-responsive transcripts across locations.

Test for Nourishment by Location Effect

We are also interested in identifying transcripts with a nourishment by location effect using all transcripts regardless of whether the transcript has already been determined to have a nourishment effect from the test above. These transcripts should have a different relationship between low and high nourishment depending on the location of the sample. These transcripts are identified by rejecting (FDR ≤ 0.05, [[2](#_ENREF_2)]) the null hypothesis that there is not a nourishment by location effect:

$$H_{0}:\left( \beta\tau\right)_{i=labj=low.t}=0$$

in favor of the alternative hypothesis the differential expression between nourishment levels is dependent on the location:

$$H_{0}:\left( \beta\tau\right)_{i=labj=low.t}\neq0.$$

To do this, we contrast the reduced location and nourishment effects model:

$$Y_{ijkt}=\mu_{\ldots t}+\beta_{i..t}+\epsilon_{ijkt}$$

with the full main effects and interaction effects model:

$$Y_{ijkt}=\mu_{\ldots t}+\beta_{i..t}+\tau_{.j.t}+\epsilon_{ijkt}$$

using the generalized linear model test in DESeq [[3](#_ENREF_3)]. From this test, we have a list of transcripts that have a distinct nourishment effects for the lab and field, which will be referred to as location-dependent nourishment-responsive transcripts.

These models were run in R [[4](#_ENREF_4)] following the DESeq [[3](#_ENREF_3)] workflow described for multi-factor designs using a pooled dispersion for all samples and performing two generalized linear model tests to identify 1) nourishment-responsive transcripts across locations and 2) location-dependent nourishment-responsive transcripts. We identified clusters of nourishment-responsive transcripts across locations and location-dependent using K-means clustering with the Mfuzz package [[5](#_ENREF_5)] from the Bioconductor repository [[6](#_ENREF_6)]. The lists of differentially expressed transcripts from each test and approach were then compared and visualized using Venn diagrams.

**Supplemental Results**

Of the 74,516 *P. metricus* transcripts, very few (60 = 0.08%) were identified to have nourishment main effects, whereas, more than twice that number of transcripts (132 = 0.18%) have a nourishment by location interaction effect (see Supplementary Table 1 and Additional_file_4.xlsx for the list of transcripts with nourishment effects and nourishment by location interaction effects). There were fewer (total of 192 transcripts) nourishment-responsive transcripts across location and location-dependent nourishment-responsive transcripts identified with this combined data approach compared to the number of nourishment-responsive transcripts identified with just the lab samples (described in main text, 284 transcripts). This is likely due to differences in variance – the dispersion of lab samples was calculated “per condition” whereas the dispersion of the lab and field samples combined was a “pooled” calculation.

The 33 (55%) transcripts with a significant nourishment effect are transcripts that share nourishment-response regardless of location (herein referred to as nourishment-specific transcripts). Supplemental Figure 4 displays the expression profiles of these 33 nourishment-specific transcripts, shown as clusters of genes with similar expression patterns (identified with the K-means approach). Most of the clusters show higher expression in the low nourishment samples compared to the high nourishment samples. Comparing these nourishment-specific transcripts to the list of 43 overlapping nourishment and caste DETs, we did not find any shared transcripts (Supplemental Figure 7), which may not be too surprising considering the majority of these overlapping nourishment and caste DETs were in the opposite directionality than predicted. Instead, we find that most (25 DETs, or 75.8%) of the nourishment-specific transcripts from this analysis overlap with the caste-related DETs identified by Berens et al. [[1](#_ENREF_1)]. Thus, our results from this alternative statistical analysis do still provide some support for the observation that genes related to nourishment level may also be related to caste differences.

For the 105 interaction-specific transcripts (i.e. the transcripts that were identified to have nourishment by location interaction effects but not nourishment effects) three of the four clusters shared the same strong pattern: higher expression in the low nourishment (worker-destined) compared to the high nourishment (gyne-destined) samples in the field (see Supplemental Figure 5 for the expression profiles of these interaction-specific transcripts by K-means clustering). If we focus on the lab-based contrast, three of the four clusters had higher expression in the high nourishment vs. the low nourishment group. These data show there are many DETs that show different directional patterns of expression in the two studies, similar to what we reported based on the DET list comparison in the main text. Of these interaction-specific transcripts, 48 transcripts overlap with the caste DETs and 20 are shared with the nourishment-responsive DETs, where eight are common to both the nourishment and caste DETs (Supplemental Figure 7).

Finally, there are 27 transcripts with both nourishment main effects and nourishment by location interaction effects in this model (Supplemental Figure 6), which display similar profiles to the nourishment-specific transcripts with most (four of six) clusters having higher mean expression in the low nourishment field samples. There is one cluster with higher expression in the high field samples compared to all other groups, and the other cluster has different directionality depending on the location (up-regulation in low nourishment in the field and high nourishment in the lab). Almost all (26 of 27) of the interaction transcripts are shared with the caste DETs, but there are only four common to both nourishment and caste DETs (Supplemental Figure 7).

**Supplemental Table 1:** Number of differentially expressed transcripts between nourishment levels across and dependent on location identified by DESeq.

|  |  | Nourishment-responsive across location | |  |
| --- | --- | --- | --- | --- |
|  |  | Yes | No | Total |
| Location-dependent nourishment-responsive | Yes | 27 | 105 | 132 |
|  | No | 33 | 74352 | 74384 |
|  | Total | 60 | 74456 | 74516 |

**Supplemental Figure 1:** Heat map of relative expressions (sample read counts scaled by library size then across each gene) for the 43 transcripts that were differentially expressed for both caste and nutrition. Transcripts are clustered based on relative expression across all samples (left). Transcripts that are up-regulated in high nutrition are highlighted in yellow, and low nutrition up-regulated transcripts are indicated by the blue color. Up-regulation in queen caste is indicated by the orange color, and transcripts that are up-regulated in worker caste is highlighted by the purple color.


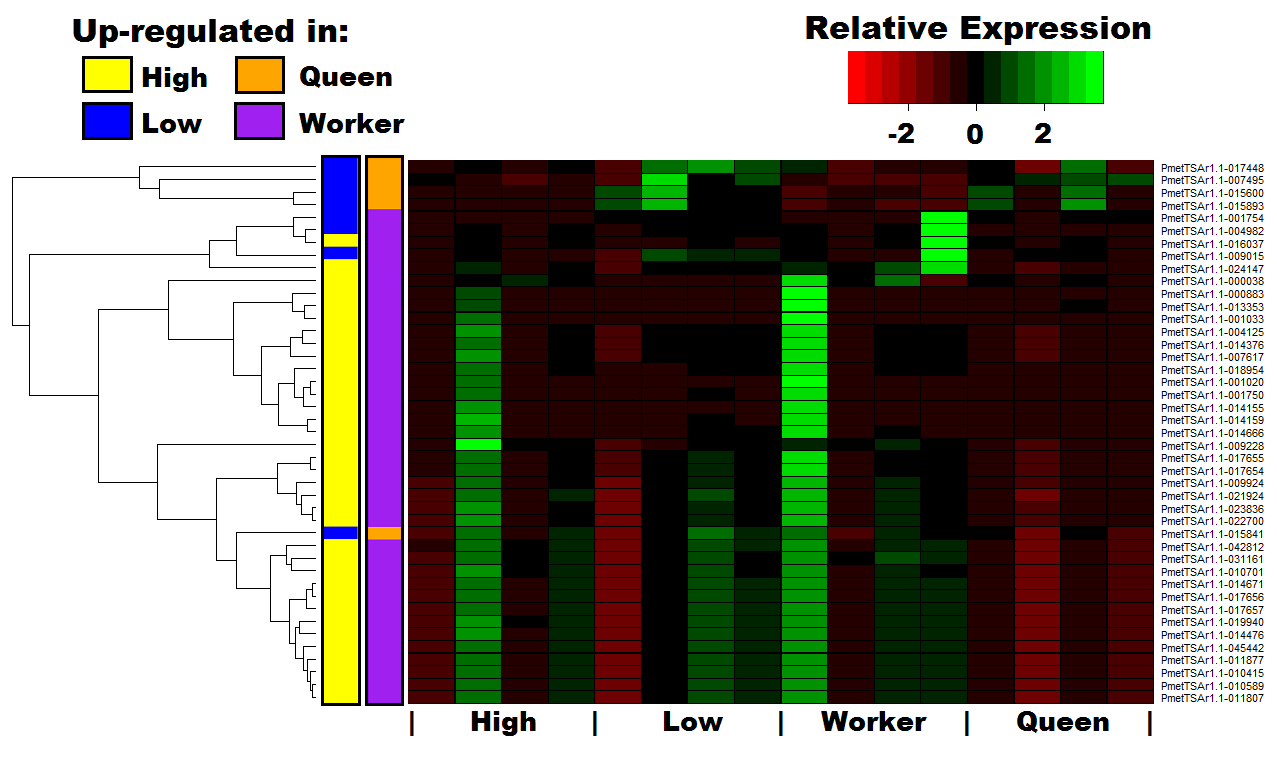


**Supplemental Figure 2:** Bar chart of GO categories significantly enriched (FDR < 0.05; one-tail) between caste differentially expressed transcripts (DETs) and remaining transcriptome. Note that all GO categories were over expressed in the caste DETs compared to the rest of the transcriptome. 17 significantly enriched GO categories were shared in common for both caste and nutrition. Directionality is indicated for the enriched GO categories and defined as the treatment group with the greater number of up-regulated DETs per category. All shared enriched GO terms are up-regulated in the worker caste.


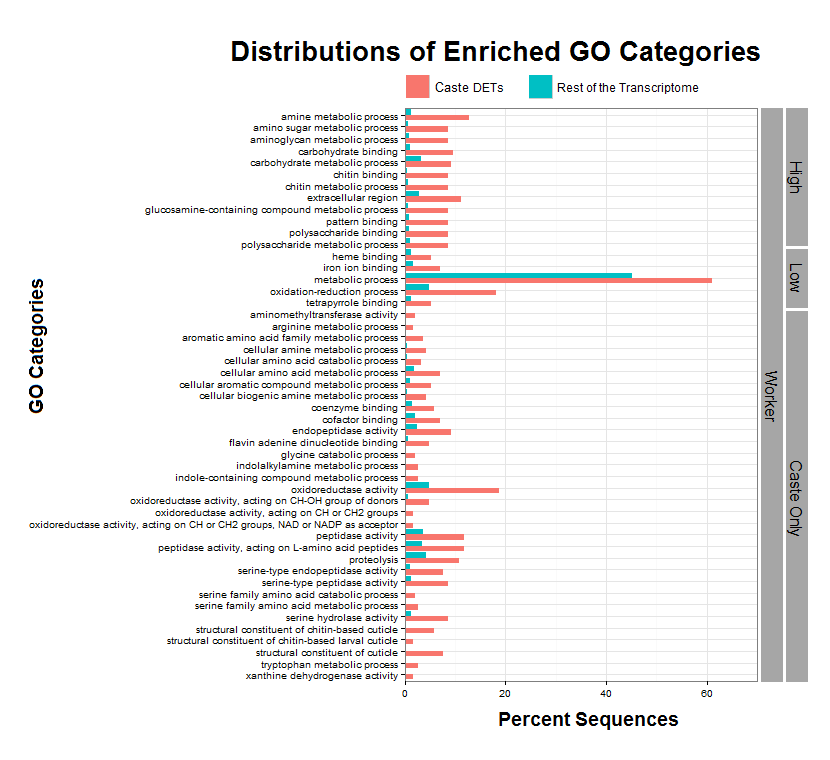


**Supplemental Figure 3:** Bar charts of the log_2_ fold changes for 23 genes from [[7](#_ENREF_7)]and orthologous transcripts (best BLAST hits to genes in [[7](#_ENREF_7)]) in this study. A positive log_2_ fold change indicates higher expression in low nutrition samples. For some genes, there were multiple best BLAST hits to transcripts in this study, so all were used for downstream analyses. From Daugherty et al. [[7](#_ENREF_7)], six genes (*ILPs*, *Kul*, *SPARC*, *tun*, *usp*, *Vg1*) were identified as up-regulated in high nutrition samples, and 4 genes (*CG11971*, *InR2*, *sNPFR, Tachykinin*) were identified as up-regulated in low nutrition. None of the orthologous transcripts in this study are differentially expressed between nutrition levels; however, there is a significant positive correlation in log_2_ fold changes between these two studies (Spearman ρ: 0.54, p-value = 0.001).


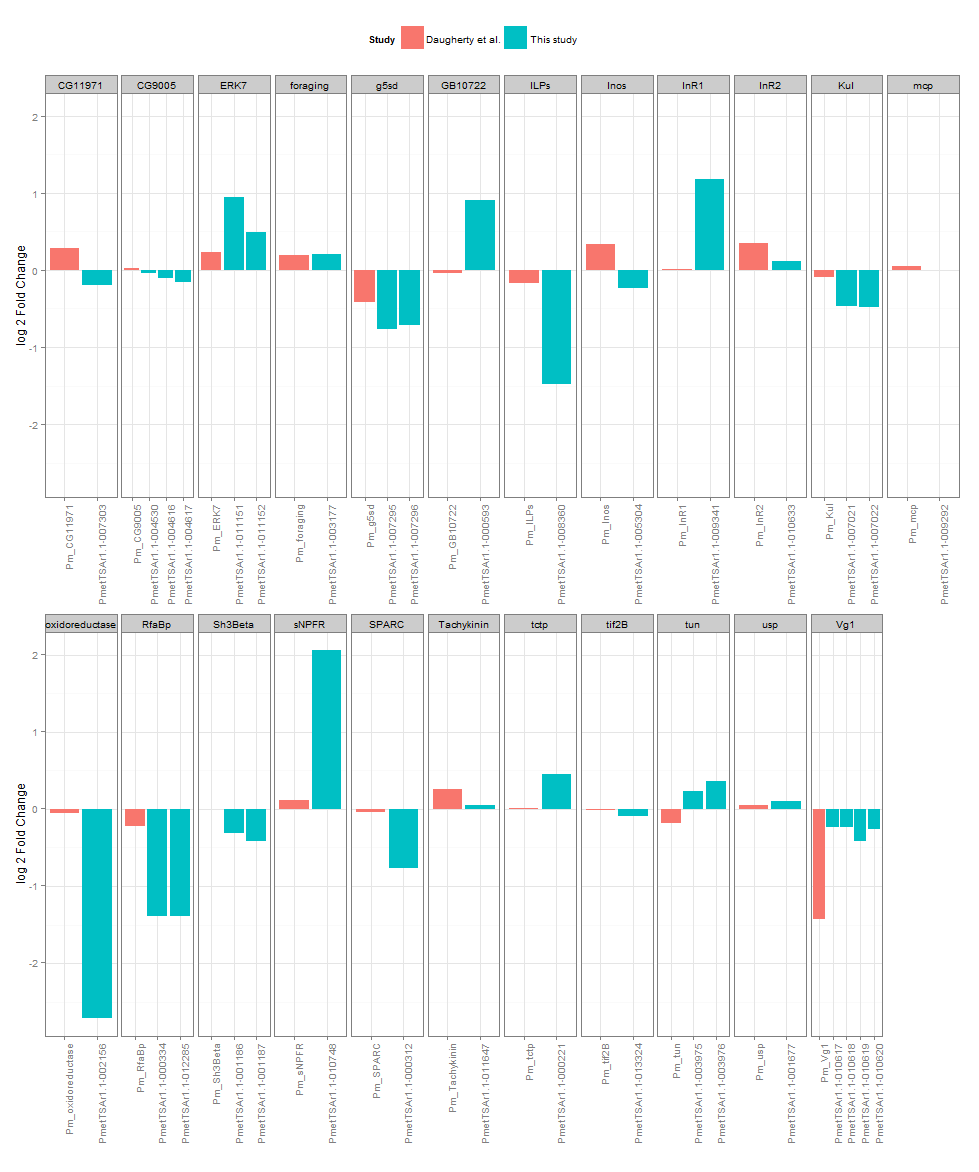


**Supplemental Figure 4:** Clustering of the nourishment-specific mean normalized expression profiles by transcript for wasps raised under high nourishment in the field, high nourishment in the lab, low nourishment in the field, and low nourishment in the lab. The number of transcripts per cluster is listed next to each of the six clusters. For each cluster, transcript normalized expression profiles are displayed as grey lines, and the mean expression profile for all transcripts within the cluster is the thick colored line.


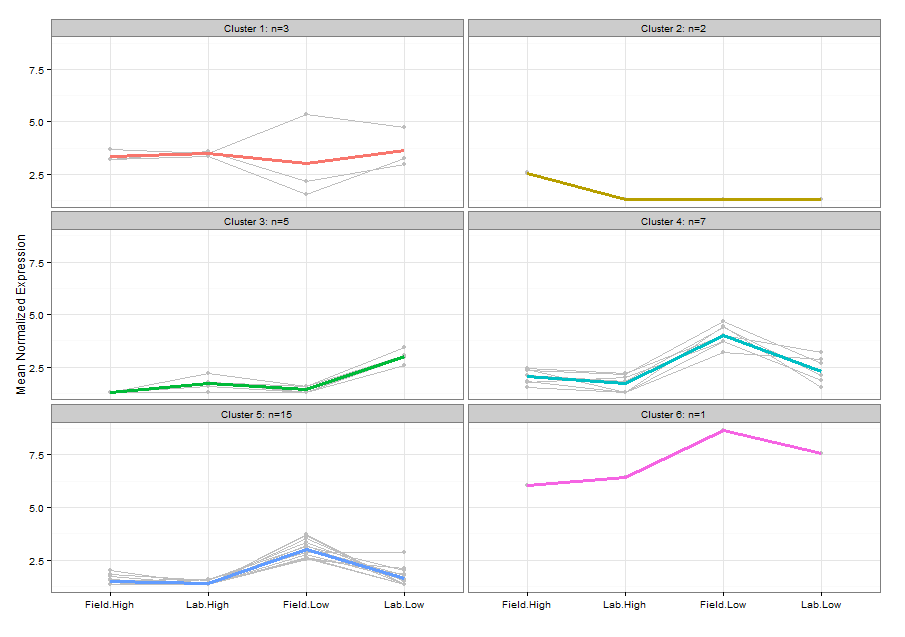


**Supplemental Figure 5:** Clustering of the interaction-specific mean normalized expression profiles by transcript for wasps raised under high nourishment in the field, high nourishment in the lab, low nourishment in the field, and low nourishment in the lab. The number of transcripts per cluster is listed next to each of the six clusters. For each cluster, transcript normalized expression profiles are displayed as grey lines, and the mean expression profile for all transcripts within the cluster is the thick colored line.


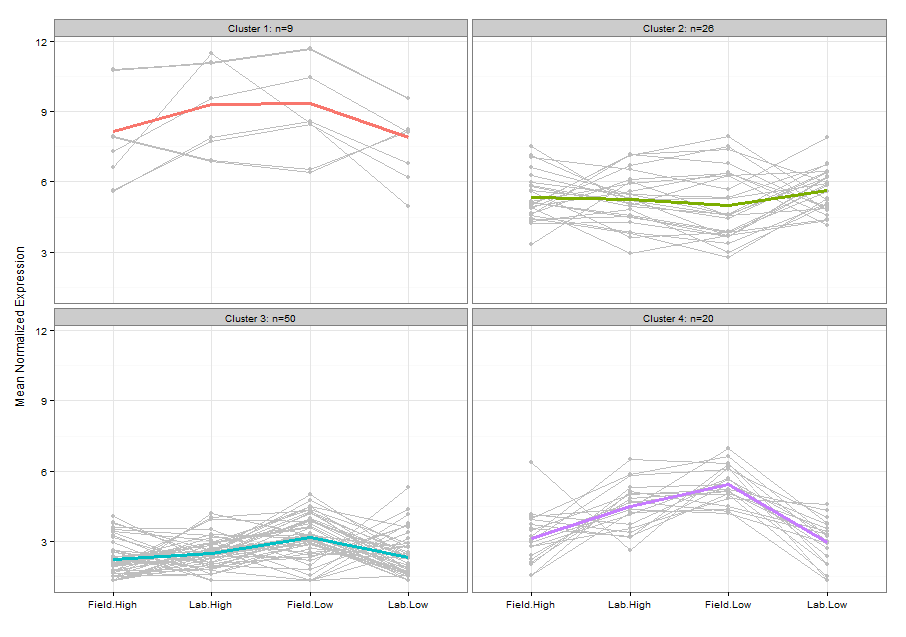


**Supplemental Figure 6:** Clustering of the mean normalized expression profiles of the 27 overlapping nourishment and interaction significant transcripts for wasps raised under high nourishment in the field, high nourishment in the lab, low nourishment in the field, and low nourishment in the lab. The number of transcripts per cluster is listed next to each of the six clusters. For each cluster, transcript normalized expression profiles are displayed as grey lines, and the mean expression profile for all transcripts within the cluster is the thick colored line.


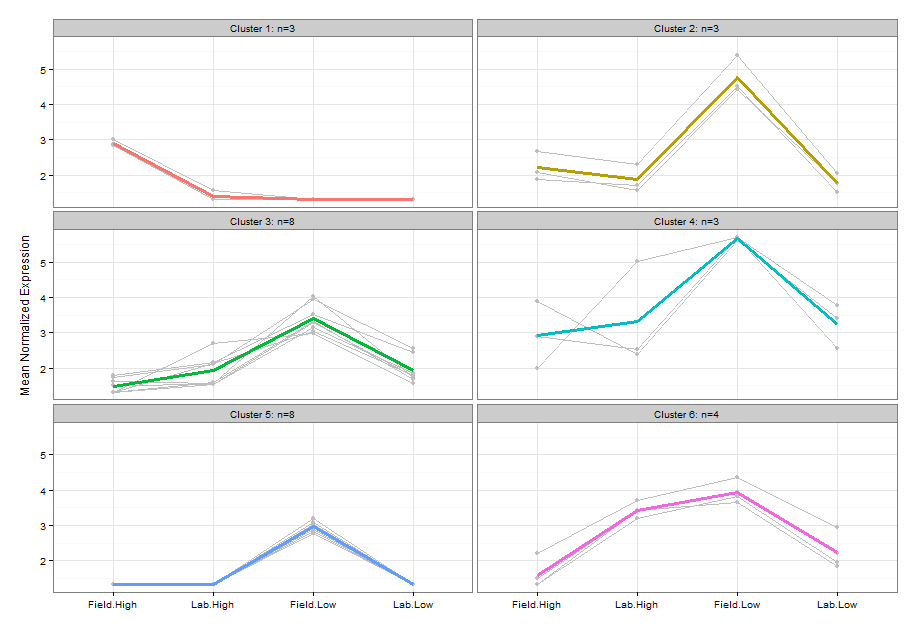


**Supplemental Figure 7:** Venn diagram of the number of transcripts identified by the modeling and list comparison approaches. For the modeling approach, all data from the lab and field were used to identify nourishment main effects and nourishment by location interaction effects using a generalized linear modeling test in DESeq. With the list comparison approach, we identified nourishment-responsive and caste-related transcripts using either the lab or field dataset, respectively.

**
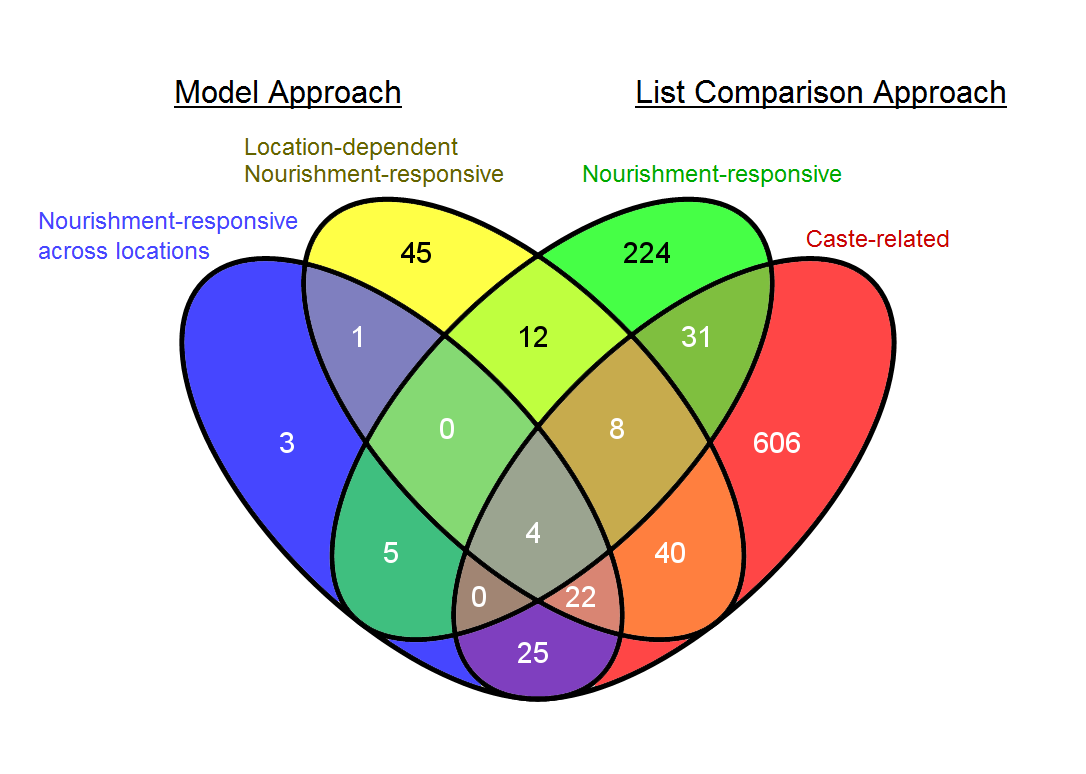
**

**References**

1. Berens AJ, Hunt JH, Toth AL: **Comparative transcriptomics of convergent evolution: Different genes but conserved pathways underlie caste phenotypes across lineages of eusocial insects.** *Molecular biology and evolution* 2014.

2. Benjamini Y, Hochberg Y: **Controlling the False Discovery Rate - a Practical and Powerful Approach to Multiple Testing.** *J Roy Stat Soc B Met* 1995, **57:**289-300.

3. Anders S, Huber W: **Differential expression analysis for sequence count data.** *Genome biology* 2010, **11:**R106.

4. R Core Team: **R: A language and environment for statistical computing.** Vienna, Austria; 2013.

5. Kumar L, M EF: **Mfuzz: a software package for soft clustering of microarray data.** *Bioinformation* 2007, **2:**5-7.

6. Edgar R, Domrachev M, Lash AE: **Gene Expression Omnibus: NCBI gene expression and hybridization array data repository.** *Nucleic Acids Res* 2002, **30:**207-210.

7. Daugherty THF, Toth AL, Robinson GE: **Nutrition and division of labor: Effects on foraging and brain gene expression in the paper wasp Polistes metricus.** *Mol Ecol* 2011, **20:**5337-5347.
